# Enhancement of the Soluble Form of OX40 and OX40L Costimulatory Molecules but Reduction of the Membrane Form in Type 1 Diabetes (T1D)

**DOI:** 10.1155/2019/1780567

**Published:** 2019-08-01

**Authors:** Jingnan An, Sisi Ding, Sicheng Li, Lili Sun, Xin Chang, Ziyi Huang, Bin Zhou, Chen Fang, Cuiping Liu, Xueguang Zhang

**Affiliations:** ^1^Jiangsu Institute of Clinical Immunology, First Affiliated Hospital of Soochow University, Suzhou, China; ^2^Institute of Blood and Marrow Transplantation, First Affiliated Hospital of Soochow University, Suzhou, China; ^3^Jiangsu Key Laboratory of Clinical Immunology, First Affiliated Hospital of Soochow University, Suzhou, China; ^4^Department of Endocrinology, Second Affiliated Hospital of Soochow University, Suzhou, China

## Abstract

This study analyzed the expression of membrane OX40 and OX40L (mOX40 and mOX40L) and levels of soluble OX40 and OX40L (sOX40 and sOX40L) in T1D patients to determine their clinical significance. Peripheral blood (PB) was collected from patients with T1D and healthy control participants. Expression of mOX40 and mOX40L on immune cells was detected by flow cytometry. Levels of sOX40 and sOX40L in sera were measured by ELISA. We demonstrated for the first time enhanced sOX40 and sOX40L expression and reduced mOX40 and mOX40L levels in T1D patients which correlated with the clinical characteristics and inflammatory factors. These results suggest that OX40/OX40L signal may be promising biomarkers and associated with the pathogenesis of T1D.

## 1. Background

Type 1 diabetes (T1D) is a chronic and organ-specific autoimmune disease, is influenced by inherited or environmental factors, is increasing in all age groups, especially examined in children, and leads to the loss of insulin production in beta cells when immune cells invade the pancreatic islets [1–3]. The activation of T cells is mediated by antigen stimulation through T cell receptors and some costimulatory molecules. It has been suggested that CTLA-4 and PD-1 contribute to the development of T1D [4–6]. Multiple clinical immune intervention trials have been provided for the treatment of T1D such as blocking the costimulation of T cells [7–9]. However, the contributions of OX40 and OX40L to the development of T1D remain to be studied.

OX40/OX40L is a pair of important positive costimulatory signal molecules in the second signal system of T cells. OX40 (CD134) and OX40L (CD252), members of the TNF receptor superfamily (TNFRSF) and TNF superfamily (TNFSF), play important roles in T cell expansion in tumors, during infectious inflammation, and in autoimmune diseases [10, 11]. OX40 is expressed in activated T cells [12], while OX40L is mainly expressed in B lymphocytes, dendritic cells, and macrophage cells [12]. OX40/OX40L signaling acts as a key role in the development and differentiation of some immunological cells, especially T cells [13, 14]. Numerous studies have shown the important role of costimulatory molecules in T1D. Blocking costimulatory interactions such as CD28/B7 or CD40/CD40L might be an effective therapy way in NOD mice [15, 16]. Blocking OX40/OX40L interactions at the late age of disease may also suppress diabetes progression [17].

The level of sOX40 and sOX40L is elevated in some tumors and infectious diseases. This suggests that sOX40 and sOX40L act as costimulators to induce immune responses. However, to the best of our knowledge, the significance of membrane and soluble forms of OX40 or OX40L expression and their correlation with clinical parameters in T1D have not been investigated. To characterize OX40 and OX40L expression and determine the role of OX40/OX40L signal in the development and pathogenesis of T1D, we evaluated the expression of sOX40 and sOX40L in the PBMC and sera of patients with T1D and healthy controls (HCs) and analyzed the correlation with clinical and inflammatory indicators.

## 2. Materials and Methods

### 2.1. Patients and Controls

In our study, blood samples were collected after overnight fasting from patients (*n* = 119) at the Endocrinology Department in the Second Affiliated Hospital of Soochow University, Suzhou, China, between 2015 and 2017. The diagnostic criteria for T1D were according to “The T1D Exchange Clinic Registry” [18]. Samples from healthy blood donors (*n* = 108) were matched to T1D patients with age, gender, and race. In addition, HCs were tested for glucose levels, all T1D autoantibody negative. The study participants were excluded if they had one of the following conditions: acute or chronic inflammatory diseases, infectious diseases, and cancer. For the detection of membrane forms of OX40 and OX40L on immune cells, 46 patients and 44 HCs were enrolled. The approval from the Ethics Review Board of the Second Affiliated Hospital of Soochow University was granted to the patients.

### 2.2. Antibodies and Flow Cytometry

To assess the expression of mOX40 in CD3^+^, CD4^+^, or CD8^+^ T cells and mOX40L in CD14^+^ monocyte and CD19^+^ B cells, peripheral blood mononuclear cell (PBMC) was isolated from T1D patients or healthy donors. Flow cytometry was performed on PBMC incubated with fluorochrome-labeled monoclonal antibodies (mAbs) for 30 min. Anti-CD3-FITC (clone: UCHT1), anti-CD4-FITC (clone: RPA-T4), anti-CD8-FITC (clone: SFCI21), anti-CD14-FITC (clone: RMO52), and anti-CD19-FITC (clone: J3-119) mAbs were from Beckman Coulter. Anti-OX40-PE (clone: ACT35) and anti-OX40L-PE (clone: RM134L) were from eBioscience. Cells were washed and then immediately measured by flow cytometry (Beckman Coulter, CA). Data were analyzed using FlowJo software (Tree Star, OR).

### 2.3. Soluble OX40 and OX40L Measurement

The samples were centrifuged at 800xg for 8 min, and the sera was stored at -80°C for ELISA. To determine the relationship of sOX40 or sOX40L with the disease activity, serum levels of sOX40 and sOX40L were determined by ELISA using kits obtained from Kalang (Shanghai) and Cusabio (Wuhan), respectively. Sera samples were plated into the 96-well microplates, and ELISA was conducted following the manufacturer's instructions. Plates were read in a microplate reader (Bio-Rad, CA) for the absorbance at 450 nm.

### 2.4. Cytokine Production

Cytokines interleukin IL-2, IL-4, IL-6, and IL-10, interferon gamma (IFN-*γ*), tumor necrosis factor-alpha (TNF-*α*), and IL-17a were quantified in patients using a cytometric bead array system (CBA) (BD Pharmingen, CA) according to the instructions of the manufacturer. For each reaction, 50 *μ*l sera were mixed with 50 *μ*l beads to each assay tube. 50 *μ*l PE detection reagent was then added, and the samples were incubated for 3 hours at room temperature. Samples were washed with wash buffer, centrifuged, and then run on flow cytometer; data were analyzed using BD™ CBA Software.

### 2.5. Statistical Analysis

Statistical analysis was performed using the IBM SPSS statistic 22 (Chicago, IL, USA). All the quantitative data was presented as the mean ± standard deviation (SD). For statistical analysis, differences in continuous variables between two independent samples were evaluated by the Mann-Whitney *U* test. Dichotomous variables were compared by the *χ* ^2^ test. Association between continuous variables was assessed by means of Spearman and partial correlation. Pearson's rank correlation analysis was used to evaluate the associations between dichotomous variables and protein levels. Covariance analysis was used. The statistical software GraphPad Prism 6.0 (GraphPad Software, La Jolla, CA) and IBM SPSS statistic 22 (Chicago, IL, USA) were used for graph creation.

## 3. Results

### 3.1. Reduced mOX40 and mOX40L Expression in T1D Patients

**Figure 1:**
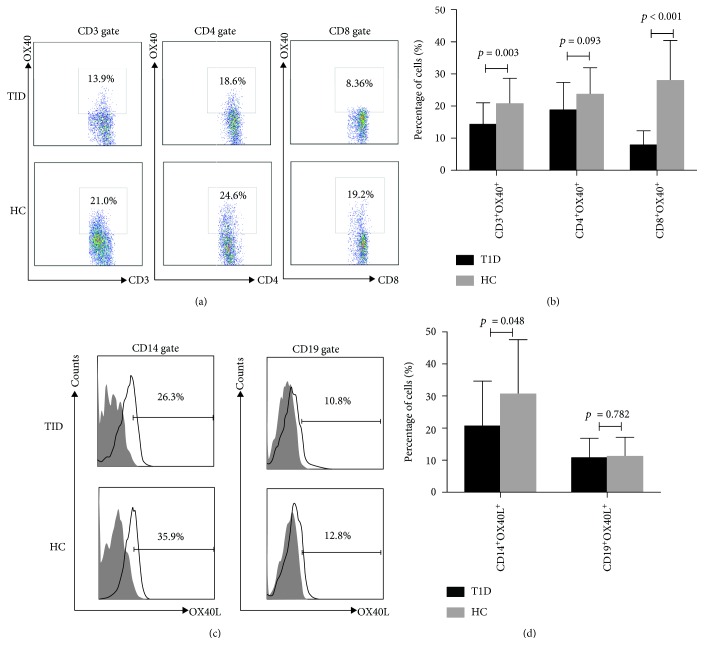
Expression of mOX40 and its ligand mOX40L in the PBMC of T1D patients. (a) The expression level of mOX40 was detected by flow cytometry of CD3^+^, CD4^+^, and CD8^+^ T cells from T1D patients and HCs. (b) Percentages of CD3^+^OX40^+^, CD4^+^OX40^+^, and CD8^+^OX40^+^ cells in the PBMC samples of patients with T1D and HCs. (c) The expression level of mOX40L was detected by flow cytometry of monocyte cells from T1D patients and HCs. CD14 and CD19 were used as markers of monocytes and B cells. (d) A histogram representative of a set of CD14^+^OX40L^+^ and CD19^+^OX40L^+^ is shown in the right figure.

The clinical characteristics of the study population are summarized in Table 1. To investigate the expression of mOX40 and mOX40L in T1D patients, blood specimens were collected from 46 T1D patients and 44 HCs. Flow cytometry analyses demonstrated that OX40 expression on CD3^+^, CD4^+^, and CD8^+^ T cells in PBMC samples was less frequent in patients with T1D than HCs (14.34 ± 1.02% vs. 22.47 ± 1.87%, *p* = 0.003; 18.78 ± 1.31% vs. 24.85 ± 1.87%, *p* = 0.093; and 7.94 ± 0.66% vs. 15.9 ± 1.87%, *p* < 0.001; respectively) (Figures 1(a) and 1(b)). Furthermore, the expression of OX40L on CD14^+^ monocytes was also decreased significantly in PBMC samples of patients with T1D compared to HCs (28.73 ± 3.69% vs. 38.87 ± 5.46%, *p* = 0.048). However, there was no significant difference in mOX40L on CD19^+^ B cells between T1D patients and HC (10.76 ± 0.93% vs. 12.02 ± 1.62%, *p* = 0.782) (Figures 1(c) and 1(d)).

### 3.2. Correlation between mOX40 and mOX40L Positive Cells and Clinical Parameters of T1D

To determine whether the negative association of mOX40 and mOX40L expression is related to T1D disease activity, we examined the relationship between the presence of mOX40 and mOX40L and the disease state. The statistical analyses of the correlation between clinical features and the expression of mOX40 and mOX40L are presented in Table 2. We found that the expression of glutamic acid decarboxylase (GAD) was negatively correlated with CD8^+^OX40^+^ T cells (*r* = −0.3996, *p* = 0.0431) and CD19^+^OX40L^+^ B cells (*r* = −0.3789, *p* = 0.0463). A significant negative correlation between CD3^+^OX40^+^ T cells (*r* = −0.3911, *p* = 0.0244), CD4^+^OX40^+^ T cells (*r* = −0.4072, *p* = 0.0187), and creatinine (Cr) was observed; we also found a significant negative correlation between CD3^+^OX40^+^ T cells (*r* = −0.4357, *p* = 0.0113) and CD4^+^OX40^+^ T cells (*r* = −0.4231, *p* = 0.0142) with uric acid (UA). However, we did not found any correlation between CD8^+^OX40^+^ T cells and Cr or UA (Supplement Figure 1).

### 3.3. Higher Serum Levels of sOX40 and sOX40L in T1D Patients

**Figure 2:**
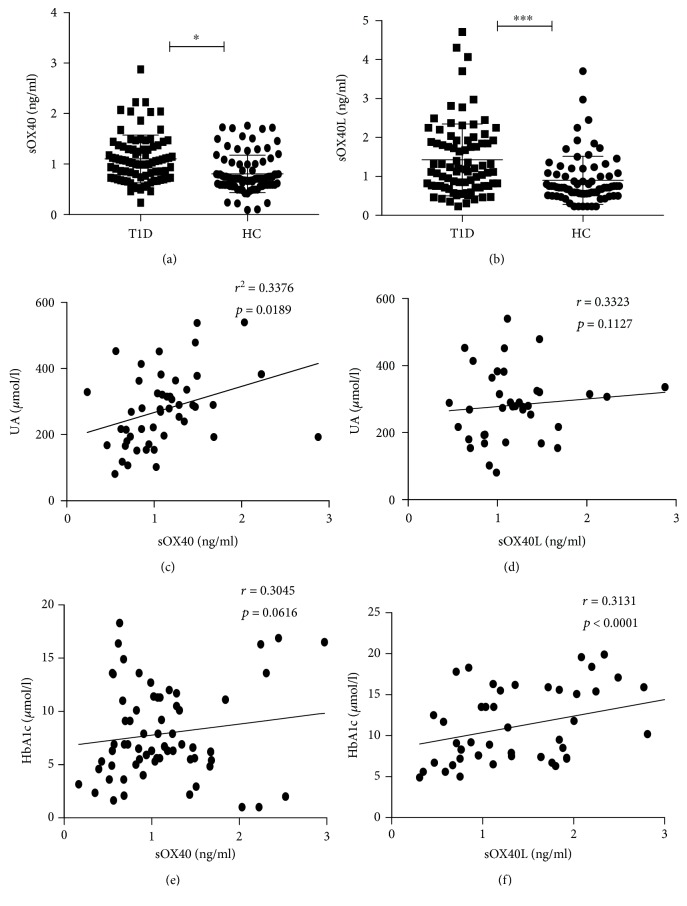
Correspondence correlation between sOX40 and sOX40L levels in HC blood and T1D disease. (a) Increased sOX40 was observed in the sera from T1D patients as compared to HCs. (b) Increased sOX40L levels were observed in the sera from patients with T1D compared with the HC group. (c) Positive correlation between sOX40 and the expression of UA. (d) No significant correlation between sOX40L and the expression of UA. (e) No significant correlation between sOX40 and the expression of HbA1c. (f) Positive correlation between sOX40L and the expression of HbA1c.

**Figure 3:**
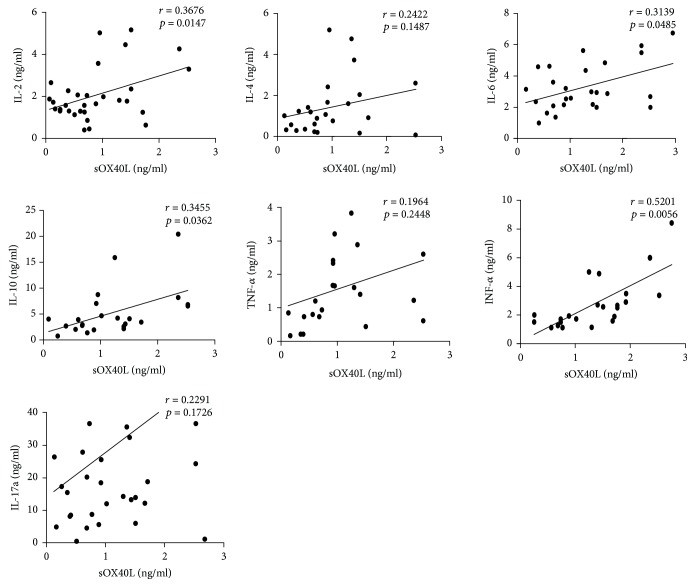
Correlation between sOX40 and sOX40L expression and sera cytokine levels in T1D patients. Positive correlation between sOX40L and the expression of IL-2, IL-6, IL-10, and IFN-*γ*.

Our previous work demonstrated that in addition to mOX40 and mOX40L, sOX40 and sOX40L are also present in human sera. Serum levels of sOX40 and sOX40L were determined by ELISA. As shown in Figure 2(a), the concentration of sOX40 in the sera of T1D patients was significantly higher than that of HCs (1.08 ± 0.06 ng/ml vs. 0.83 ± 0.08 ng/ml, *p* = 0.042). Meanwhile, we found that the concentration of sOX40L was also significantly higher than that in HCs (1.42 ± 0.10 ng/ml vs. 0.83 ± 0.08 ng/ml, *p* < 0.0001) (Figure 2(b)). To explore whether sOX40 and sOX40L are involved in the development of T1D, we further analyzed the relationship between sOX40, sOX40L, and disease activity. We conducted a correlation analysis and found a positive correlation between sOX40 expression and UA (*r* = 0.3376, *p* = 0.0189) and hemoglobin A1c (HbA1c) (*r* = 0.3045, *p* = 0.0616), that is, T1D patients with higher sOX40 expression levels had higher UA and HbA1c (Figures 2(c) and 2(e)). The correlation between sOX40L and hemoglobin A1c (*r* = 0.3131, *p* < 0.0001) was also positive but was not significant with UA (*r* = 0.3323, *p* = 0.1127) (Figures 2(d) and 2(f)). These data suggest that the expression of sOX40 and sOX40L correlates with several parameters of disease activity. To evaluate whether OX40 and OX40L play a role in the process of T cell differentiation, we measured the serum levels of some cytokines such as IL-2, IL-4, IL-6, IL-10, IFN-*γ*, TNF-*α*, and IL-17a in T1D patients, finding no significant correlation between sOX40 expressions and cytokines (Table 3). However, sOX40L expression in T1D patients was positively correlated with serum levels of IL-2 (*r* = 0.3676, *p* = 0.0147), IL-6 (*r* = 0.3139, *p* = 0.0485), IL-10 (*r* = 0.3455, *p* = 0.0362), and IFN-*γ* (*r* = 0.5201, *p* = 0.0056) (Figure 3).

### 3.4. Negative Correlation between Serum Levels of sOX40 and mOX40 in T1D Patients

**Figure 4:**
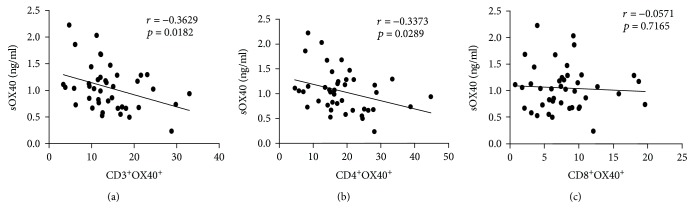
Correlation between serum levels of sOX40 and mOX40 in T1D patients. (a) Significant negative correlation between sOX40 and mOX40 expression on CD3^+^ T cells. (b) Significant negative correlation between sOX40L and mOX40 expression on CD4^+^ T cells. (c) Negative correlation between sOX40L and mOX40 expression on CD8^+^ T cells.

Correlation analyses demonstrated that there was a significant negative association between serum OX40 levels and mOX40 expression on CD3^+^ T cells (*r* = −0.3629, *p* = 0.0182) and CD4^+^ T cells (*r* = −0.3373, *p* = 0.0289) in T1D patients (Figure 4). Although not significant, there was a negative association between sOX40 levels and mOX40 expression on CD8^+^ T cells (*r* = −0.0571, *p* = 0.7186) (Table 3).

## 4. Discussion

Costimulatory molecules play important roles in regulation of the immune response in autoimmune diseases. Therefore, they may be useful as treatment indicators for the disease [19].

The TNF/TNFR family members of costimulatory molecules are critical in regulating T cell responses [20]. Here, we detected the expression of membrane and soluble forms of OX40 and OX40L on immune cells in T1D patients and determined their clinical significance. For the first time, we detected the membrane and soluble forms of OX40 and OX40L expression in the PBMC of T1D patients and analyzed the correlation with disease activity. We found that sOX40 and sOX40L expression in T1D patients was significantly higher compared with the HCs. However, the expression of mOX40 and mOX40L in T1D patients was significantly lower compared with that in HCs. There was no significant correlation of mOX40L of CD19^+^ B cells in T1D patients and HCs. In addition, sOX40 levels in patients were positively correlated with the disease activity, as indicated by T1D disease activity, and sOX40L levels in the patients were positively correlated with inflammatory cytokines. Our findings suggest that sOX40 and sOX40L might counteract the aberrant immune response and potentially serve as monitoring indicators of disease progression and therapeutic targets in T1D treatment.

Costimulatory molecules can induce the activation of T cells, promote cell survival, support the formation of potential memory T cells, and produce the release of the cytokines [21, 22]. Therefore, the costimulatory molecules may be useful for the diagnosis and treatment of some diseases. Both membrane and soluble forms of costimulatory molecules play important roles in the regulation of immune networks [23]. Costimulatory molecules with soluble forms could be generated from proteolytic cleavage, such as PD-L1, B7H3 [24], ICOS, and ICOSL [25], or could be generated from mRNA splicing like CTLA-4 [26] and PD-1 [27]. It has been found that sOX40 is expressed in patients with amyotrophic lateral sclerosis [28] and chronic lymphocytic leukemia [29], while sOX40L is expressed in the sera of patients with acute coronary syndrome, bronchial asthma (adult), Henoch-Schonlein purpura (children), and rheumatoid arthritis [30–33]. In our study, we observed relatively higher sOX40 and sOX40L levels but lower mOX40 and mOX40L in T1D patients, suggesting that sOX40 and sOX40L might have regulatory functions opposite to mOX40 and mOX40L. High levels of soluble molecule may be the consequence of two different processes, such as high production or decreased depletion. In T1D patients, we speculate that soluble levels increase because mOX40 and mOX40L are cleavaged into the soluble forms, leading to higher sOX40 and sOX40L expression in PBMC compared with the HCs.

Autoantibodies in T1D are risk indicators for the diagnosis and prediction of the disease and surrogate markers for autoimmune diabetes [34–37]. The presence of several autoantibodies such as islet cell autoantibody (ICA) and GAD indicates an autoimmune pathogenic response to beta cells [38]. We measured the membrane variants of OX40 and OX40L in T1D individuals, which were reduced compared to HC. Meanwhile, GAD expression is also associated with mOX40 and mOX40L expression. However, some other indicators such as higher levels of UA are also associated with enlarged risk of the clinical manifestations of diabetic nephropathy in persons with T1D [39, 40]. Moreover, HbA1c is the most common and widely accepted indicator of T1D [41–43]. In our study, we found that T1D patients with higher sOX40 expression levels had higher UA, and higher sOX40L expression levels had higher HbA1c, which means OX40 and OX40L are promising markers for T1D.

The detailed functions of some costimulatory molecules are still controversial. Until now, one of the most attractive approaches to prevent T1D is using islet antigen-specific regulatory T cells (Tregs). Luczynski et al. discovered that the mRNA level of OX40 was lower in Treg cells of children with T1D when compared to the reference patients [2]. However, Szypowska et al. observed that T1D children had higher frequency of CD4^+^CD25^high^OX40^+^ cells than healthy subjects [44]. In our study, we found a reduction of the membrane form of OX40 on CD3^+^, CD4^+^, and CD8^+^ T cells and a reduction of OX40L on CD14^+^ monocytes in T1D patients correlated with clinical parameters. Bresson et al. have found that OX40 agonist therapy can slow down T1D progression [45]. We speculate that increasing the level of OX40 may be a new therapeutic strategy.

## 5. Conclusions

Taken together, our study revealed the dissociation between mOX40 and mOX40L expression and sOX40 and sOX40L levels in T1D patients for the first time. We provided evidence for the diagnostic value of OX40 and OX40L in T1D patients. The abnormal expression of OX40 and OX40L molecules appears to be connected with the severity of the disease. Thus, OX40 and OX40L may be promising biomarkers for diagnosis and prognosis of T1D. The in-depth mechanisms of membrane and soluble OX40 and OX40L in T1D remain to be elucidated, and the role of OX40 and OX40L in immune pathogenesis of T1D requires further research.

## Figures and Tables

**Table 1:** Clinical features of the study population.

	Type 1 diabetes (*n* = 119)	Healthy controls (*n* = 108)	*p* value^a^
Age (years)	28.5 (10-53)	29.4 (24-40)	0.75
Gender, female (*n*%)	57 (47.9%)	51 (46.8%)	0.97
Duration (years)	7 (0-39)	—	—
Fasting venous blood glucose (mmol/l)	11.9 (3.99-22.3)	5.2 (3.8-6.4)	0.01
Cr (*μ*mol/l)	54.2 (26-98)	82.3 (44-112)	0.04
BUN (mmol/l)	4.4 (1.4-8.4)	—	—
UA (*μ*mol/l)	275.7 (81-540)	—	—
ALT (IU/l)	18.9 (2-92)	—	—
AST (IU/l)	20.0 (8-62)	—	—
TC (mmol/l)	4.4 (0.8-7.6)	—	—
TG (mmol/l)	1.1 (0.4-5.7)	—	—
LDL (mmol/l)	2.1 (1.1-5.4)	—	—
HDL (mmol/l)	1.4 (0.7-2.4)	—	—
Fasting C-peptide (ng/ml)	1.6 (<0.01-35)	—	—
Anti-ICA positive (%)^b^	11 (27%)	—	—
Anti-GAD positive (%)^b^	17 (41%)	—	—
Anti-IAA positive (%)^b^	3 (7%)	—	—
HbA1c (%)	9.12% (5.3-17.1%)	—	—
Ketosis	17 (41%)	—	—
IFN-*γ* (pg/ml)	1.5 (0-17.2)	—	—
IL-2 (pg/ml)	2.7 (0.4-11.9)	—	—
IL-4 (pg/ml)	2.1 (0-14.1)	—	—
IL-6 (pg/ml)	4.2 (0-15.6)	—	—
IL-10 (pg/ml)	4.7 (0.7-20.4)	—	—
TNF-*α* (pg/ml)	3.1 (0-17.2)	—	—
IFN-*γ* (pg/ml)	3.1 (0.4-19.3)	—	—
IL-17a (pg/ml)	26.0 (0-159.9)	—	—

^a^ *p* value is based on the statistical analysis by the Mann-Whitney *U* test or the chi-square test assessing overall group differences. ^b^Not all the patients have the antibody results, only 52/119 patients received an autoantibody screening. ^c^Abbreviations: Cr: creatinine; BUN: urea; UA: uric acid; ALT: alanine aminotransferase; AST: aspartate transferase; TC: cholesterol; TG: triglycerides; LDL: low-density lipoprotein; HDL: high-density lipoprotein; IAA: insulin autoantibody.

**Table 2:** Correlation between clinical features and membrane levels of OX40 and OX40L.

	CD4^+^OX40^+^		CD8^+^OX40^+^		CD3^+^OX40^+^		CD19^+^OX40L^+^		CD14^+^OX40L^+^	
	*r*	*p*	*r*	*p*	*r*	*p*	*r*	*p*	*r*	*p*
Ketosis	0.2692	0.2257	0.1746	0.4301	0.2770	0.2121	-0.3000	0.1750	0.1307	0.5619
GAD	-0.0570	0.7818	-0.3996	0.0431	-0.0571	0.7816	-0.3789	0.0463	-0.1401	0.4948
ICA	-0.0121	0.9529	-0.1582	0.4401	-0.0365	0.8593	-0.0608	0.7677	0.1245	1.0042
IAA	0.1467	0.4746	-0.1467	0.4746	0.1735	0.3968	-0.0133	0.9485	-0.0933	0.6502
DKA	0.4468	0.0371	0.1043	0.6443	0.3725	0.0878	-0.0744	0.7419	-0.0148	0.9475
HDL	-0.3474	0.0555	-0.2594	0.1588	-0.3528	0.0516	-0.1488	0.4245	-0.1671	0.3689
LDL	-0.1773	0.3401	-0.1264	0.4979	-0.2231	0.2276	-0.1470	0.4300	-0.1408	0.4501
TC	-0.2280	0.2019	-0.2848	0.1081	-0.2739	0.1230	-0.1321	0.4638	-0.1053	0.5597
TG	0.3627	0.0380	0.0844	0.6404	0.2936	0.0973	0.2199	0.2189	0.1634	0.3637
ALT	-0.0560	0.7605	0.1015	0.5804	0.0540	0.7688	0.0421	0.8190	0.1166	0.5252
AST	0.0852	0.6426	0.2025	0.2663	0.2056	0.2591	0.0343	0.8519	0.0479	0.7943
LDH	0.0260	0.9107	0.0655	0.9978	0.1383	0.5500	0.0416	0.8576	0.1498	0.5168
GGT	-0.0510	0.8085	0.1969	0.3455	-0.1076	0.6086	0.0947	0.6522	-0.3137	0.1267
Cr	-0.4072	0.0187	-0.1674	0.9926	-0.3911	0.0244	0.0363	0.8410	-0.2899	0.1017
BUN	-0.2858	0.1069	-0.1345	0.4557	-0.2436	0.1719	0.1462	0.4170	-0.1055	0.5589
UA	-0.4231	0.0142	-0.6887	0.7033	-0.4357	0.0113	0.0885	0.6239	-0.3246	0.0653
GLU	0.1162	0.5802	-0.4501	0.8308	0.1328	0.5268	0.0076	0.9971	-0.0561	0.7897
C0	-0.1418	0.4238	-0.1189	0.5031	-0.2069	0.2404	-0.0207	0.9072	0.1722	0.3300
C120	-0.2638	0.2901	-0.1209	0.6328	-0.3487	0.1562	0.0830	0.7432	0.3595	0.1429
HbA1c	0.4666	0.0047	0.0926	0.5966	0.3930	0.0195	0.3994	0.0175	0.3332	0.0504

**Table 3:** Correlation between clinical features and soluble levels of OX40 and OX40L.

	sOX40		sOX40L	
	*r*	*p*	*r*	*p*
Ketosis	0.2191	0.7217	-0.0344	0.8395
GAD	0.0289	0.3379	0.0840	0.4954
ICA	0.0070	0.7302	0.1846	0.1318
IAA	0.0607	0.7442	0.1463	0.2337
DKA	0.0962	0.6942	-0.0107	0.9448
HDL	0.1826	0.1159	-0.0511	0.6630
LDL	0.0733	0.7493	0.1849	0.1123
TC	0.0229	0.9884	0.1528	0.1846
TG	-0.0635	0.1745	0.0131	0.9094
ALT	-0.2506	0.7772	-0.1072	0.3632
AST	-0.1859	0.6487	-0.1434	0.2228
LDH	-0.2732	0.6829	-0.0224	0.8851
GGT	-0.0967	0.7239	-0.142	0.3253
Cr	-0.0148	0.5676	-0.1343	0.2443
BUN	-0.1330	0.1402	-0.0909	0.4317
UA	0.3376	0.0189	-0.1127	0.3323
GLU	0.1428	0.3729	0.0974	0.4627
C0	0.0398	0.4693	0.1008	0.3736
C120	0.2641	0.4974	0.1211	0.4952
HbA1c	0.3045	0.0616	0.3131	0.0001
CD4^+^OX40^+^ T	-0.3373	0.0225	—	—
CD8^+^OX40^+^ T	-0.4733	0.7018	—	—
CD3^+^OX40^+^ T	-0.3629	0.0162	—	—
CD19^+^OX40L^+^ B	—	—	0.2351	0.0683
CD14^+^OX40L^+^ monocyte	—	—	0.2499	0.0521
IL-2	0.2461	0.1137	0.3676	0.0252
IL-4	0.1455	0.1232	0.2422	0.1487
IL-6	0.0214	0.0196	0.3139	0.0485
IL-10	-0.1077	0.1482	0.3455	0.0362
TNF-*α*	0.4713	0.0569	0.1964	0.2448
IFN-*γ*	0.4238	0.0922	0.5201	0.0056
IL-17a	0.4947	0.0535	0.2291	0.1726

## Data Availability

The data used to support the findings of this study are available from the corresponding authors upon request.

## Abbreviations

CBA: Cytometric bead array
GAD: Glutamic acid decarboxylase
HCs: Healthy controls
HbA1c: Hemoglobin A1c
IFN-*γ*: Interferon gamma
ICA: Islet cell autoantibody
mAbs: Monoclonal antibodies
PBMC: Peripheral blood mononuclear cells
SD: Standard deviation
T1D: Type 1 diabetes
TNFRSF: TNF receptor superfamily
TNFSF: TNF superfamily
TNF-*α*: Tumor necrosis factor-alpha
Tregs: Antigen-specific regulatory T cells
UA: Uric acid.

## Supplementary Materials

Supplementary Materials Supplement Figure 1: correlation between CD3^+^OX40^+^, CD4^+^OX40^+^, and CD8^+^OX40^+^ and clinic pathological characteristics. Negative correlation between CD3^+^OX40^+^ and CD4^+^OX40^+^ and the expression of UA and Cr. No correlation between CD8^+^OX40^+^ and the expression of UA and Cr.
