# Supplementary material for: Enhancement of the Soluble Form of OX40 and OX40L Costimulatory Molecules but Reduction of the Membrane Form in Type 1 Diabetes (T1D)
Source: J Immunol Res. 2019 Aug 1;2019:1780567. doi: 10.1155/2019/1780567 (PMC6701347; doi:10.1155/2019/1780567)
Supplement: Supplementary Materials — Supplement Figure 1: correlation between CD3+OX40+, CD4+OX40+, and CD8+OX40+ and clinic pathological characteristics. Negative correlation between CD3+OX40+ and CD4+OX40+ and the expression of UA and Cr. No correlation between CD8+OX40+ and the expression of UA and Cr. [file 1780567.f1.pdf]

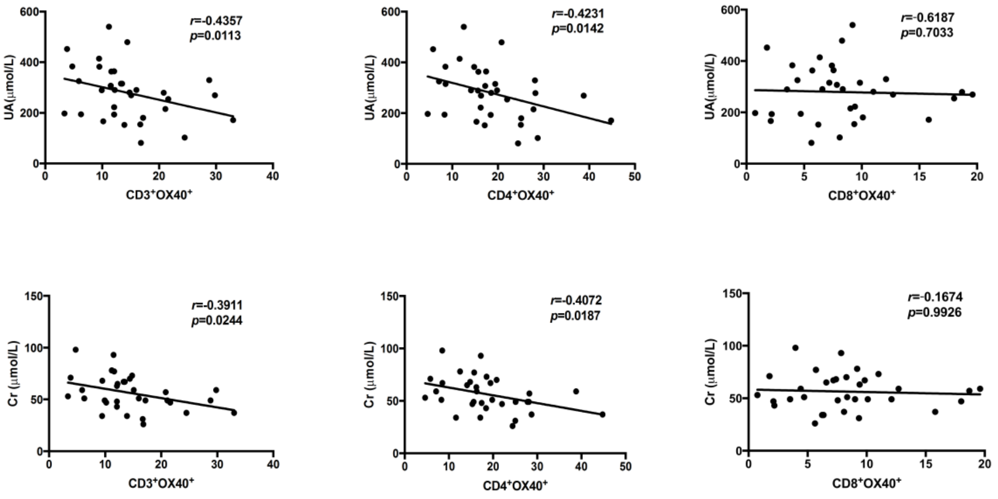

Supplement Figure.1

Supplement Figure.1 Correlations between $CD3^+OX40^+$, $CD4^+OX40^+$, $CD8^+OX40^+$ and clinic pathological characteristics. Negative correlation between $CD3^+OX40^+$, $CD4^+OX40^+$ and the expression of UA and Cr. No correlation between $CD8^+OX40^+$ and the expression of UA and Cr.
